# Good Mid-Term Implant Survival of a Novel Single-Design Rotating-Hinge Total Knee Arthroplasty

**DOI:** 10.3390/jcm12196113

**Published:** 2023-09-22

**Authors:** Matthias Schlechter, Christoph Theil, Georg Gosheger, Burkhard Moellenbeck, Jan Schwarze, Jan Puetzler, Sebastian Bockholt

**Affiliations:** 1 Department of Orthopedics and Tumor Orthopedics, Muenster University Hospital, Albert-Schweitzer-Campus 1, 48149 Muenster, Germany; dr.schlechter@icloud.com (M.S.); sebastian.bockholt@ukmuenster.de (S.B.); 2 Department of Orthopedics, St. Elisabeth Hospital Damme, Lindenstraße 3-7, 49401 Damme, Germany

**Keywords:** revision arthroplasty, revision total knee arthroplasty, total knee arthroplasty, TKA, periprosthetic joint infection

## Abstract

Background: Rotating-hinge knee (RHK) implants are an option for knee arthroplasty in cases of instability or severe bone loss. However, the revision rate can be increased compared to unconstrained implants which mandate improvements in implant design. This study investigates a novel RHK design for total knee arthroplasty (TKA). Methods: Retrospective analysis of a single design RHK TKA (GenuX MK, Implantcast) implanted between 2015 and 2019 including 133 patients (21 primary TKA, 112 revisions). The main indication for revision TKA was second-stage reimplantation following infection. The median follow-up amounted to 30 months (IQR 22–47). Results: In total, 13% (18/133) of patients underwent revision surgery after a mean time of 1 month (IQR 0–6). The main reason for the revision was (re-) infection in 8% of patients. All revisions were performed following revision TKA and none following primary TKA. There were no revision surgeries for loosening or instability. The revision-free implant survival of 83% was (95% CI 75–91%) after five years. A higher number of previous surgeries was associated with a greater revision risk (*p* = 0.05). Conclusion: Revision and complex primary TKA using a single-design RHK implant results in good implant survival at mid-term follow-up although infection remains a major concern.

## 1. Introduction

As numbers of total knee arthroplasties (TKA) and consequently associated revision surgeries show a continuous increase, rotating-hinge knee (RHK) prostheses gain importance for a variety of indications besides oncologic reconstructions [1,2,3,4,5,6]. This increase in knee revision arthroplasties is highlighted by one study that investigated the development of knee revision arthroplasty burden in a European country and found a steady increase from less than 1% of all knee arthroplasty procedures to around 4% in the last 20 years [1].

Contemporary implant designs including modular components and stems with offset adjustments as well as augments, sleeves and diverse fixation methods are frequently used in complex revision arthroplasty to manage aseptic loosening, ligamentous instabilities, bone loss, periprosthetic fracture or infection.

Historically, hinged implants in primary cases showed poor outcomes and low short-term survival due to enormous forces transmitted to the bone–cement interface causing early loosening [1,7]. Improvements in biomaterials, fixation technology, design and the axis mechanism allow for the generation of a rotation at a higher degree of mobility with a reduction in stress at the implant–bone interface at the same time. In addition, augments such as cones, sleeves and wedges with porous surfaces showed advantages in the reconstruction of bone defects and a decrease in stress transfer [2,4,8,9,10,11,12,13,14,15].

Surgeons still encounter high complication rates when using constrained TKA systems and current studies report implant survivorship of modern rotating-hinge designs to be 52–85% at 5 years [4,9] to 71–92% at 10 years [9,16]. However, one potential disadvantage of previous studies [2,3] has been the inclusion of multiple designs of RHK implants or rotating-hinge distal femoral replacements which would be an additional variable that needed to be controlled when investigating outcome and revision risk after RHK TKA.

Considering these heterogeneous results that can also be attributed to implant designs included and ongoing improvements in implant technology, the performance of new RHK designs is of interest.

The study’s purpose was to investigate implant survival rates of this single-design RHK and identify patient- and surgery-related risk factors for failure and reinfection in patients treated for PJI, as well as to evaluate joint function through postoperative Oxford Knee Score (OKS), active flexion and extension deficit.

## 2. Materials and Methods

### 2.1. Study Design

Prior to the study, ethical approval was obtained by the local ethics committee (Ethikkommission der Ärztekammer und der Westfälischen Wilhelms Universität Münster, reference number 2019-650-f-S).

We retrospectively queried our institution’s prospectively maintained database on arthroplasty surgeries and identified all TKA surgeries that were performed between May 2015 and December 2019. This period was chosen because the implant design was then introduced in our department and was the only rotating-hinge TKA implant used from then onwards. We identified 133 patients with a single-design rotating-hinge TKA (MUTARS GenuX MK, Implantcast GmbH, Buxtehude, Germany). We did not include megaprosthetic RHK implants.

We did not use any other rotating- or fixed-hinge implant designs during the study period.

### 2.2. Patients

The cohort included 21 patients who underwent primary TKA with instability or bone defects (gross ligament instability in 76% (16/21) of cases; extensive bone defects in 24% (5/21) of cases) and 112 patients with revision knee arthroplasty (second-stage reimplantation following PJI in 63% (70/112), aseptic loosening in 29% (32/112), instability in 5% (6/112) and periprosthetic fracture in 3% (4/112)). The median follow-up amounted to 30 months (IQR 22–47). Specifically, all patients who underwent surgery following second-stage reimplantation for PJI had a minimum follow-up of one year.

A total of 48% of the patients were men (64/133). The percentage of smokers was 16% (21/133) and the proportion of diabetics was 26% (35/133). Further patient demographics are given in Table 1.

### 2.3. Definitions

We defined prosthetic failure requiring revision and exchange of the implant as the primary endpoint. Secondary study endpoints were death, revision without implant revision and re-infection-free survival for patients treated for PJI.

The diagnosis of loosening was based on clinical and radiological findings as proposed by the Knee Society’s evaluation system with three radiographic views [17]. All knees underwent aspiration prior to revision surgery and infection was then diagnosed using the criteria of the Musculoskeletal Infection Society (MSIS) of 2011 [3]. After PJI, treatment success was defined based on the modified Delphi consensus criteria [18,19].

In cases of revision TKA, previous revision surgeries were analyzed and counted. For septic revision surgeries, all previous surgeries for PJI were counted including debridement and component exchanges, one-stage revisions and two-stage revisions. We extracted demographic data from the electronic patient files and calculated the body mass index (BMI), age-adjusted Charlson Comorbidity Index (CCI) [20] and the American Society of Anesthesiologists class (ASA) [21].

### 2.4. Surgical Procedures and Implant Features

Surgical access was gained through a standard medial peripatellar arthrotomy. For the revision TKA, existing components were carefully removed, and a thorough debridement was performed removing all scar tissue and in cases of PJI all infected tissue. In all revisions, a minimum of 3 to 5 microbiological samples were taken and cultured for a minimum period of 7 to 14 days depending on microbiology growth. The implant system used offers multiple reconstruction options with stems, offset couplers and wedges to reconstruct the joint line and implant position anatomically. The general approach regarding the use of cement was to perform a hybrid fixation with diaphyseal engaging cementless, hydroxyapatite-coated stems with a minimum length of 150 mm whenever possible, but stems were cemented when diminished bone quality was encountered or if only a short-stem anchorage due to ipsilateral hardware or diaphyseal deformity was achievable. In total, 6% of surgeries (8/133) were done in a fully cemented technique and 7% (9/133) had one stem cemented and one with hybrid fixation. The remaining 87% (116/133) of patients underwent hybrid fixation. In planned aseptic revisions gentamicin and clindamycin polymethylmethacrylate (PMMA) bone cement was used (Copal G+C, Heraeus medical, Wehrheim, Germany) and in cases of resistant bacteria in septic revisions gentamicin and vancomycin PMMA (Copal G+V, Heraeus medical, Wehrheim, Germany) was used for second stage reimplantation. All cases of infection underwent at least 2 weeks of tailored intravenous antibiotics and at least 6 weeks of antibiotics in total in between stages. Antibiotic suppression was not used. Postoperatively, full weight bearing and unrestricted active and passive mobilization were allowed except for cases of difficult capsular reconstruction or damage to the patella tendon in which knee flexion was limited to 30-60 degrees for a minimum of 2 weeks postoperatively.

Postoperatively, the function was assessed using the Oxford Knee Score (OKS) [22] derived from the last clinical examination at our department for all. For this score, the patients answered twelve questions regarding knee function in everyday activities from 0 to 4 points. The maximum is 48 indicating the best possible function while 0 is the lowest score indicating severe problems with different activities. Uncomplicated primary TKA usually results in an OKS > 30 [23].

### 2.5. Statistical Analysis

Data collection and statistical analysis were performed using Excel (Microsoft Corporation, Redmont, Washington, DC, USA) and SPSS Statistics for Windows Version 25 (IBM Corporation, Armonk, NY, USA). All patient records were anonymized prior to analysis.

Descriptive statistics were used to analyze the distribution of data, means and ranges were calculated for parametric data; medians and interquartile ranges (IQR) for nonparametric data. Contingency tables were analyzed using the chi^2^-test. While parametric data was analyzed using Student’s *t*-test, non-parametric analyses were performed using the Mann–Whitney-U-Test. Survival analysis was performed using the Kaplan–Meier method and differences in survival and influencing factors were assessed using the log-rank test. Statistical significance was defined as *p* ≤ 0.05.

## 3. Results

### 3.1. Revisions

In total, 13% (18/133) of patients underwent revision surgery after a mean time of 1 month (IQR 0–6). Reasons for revision were (re-)infection in 4% (6) of patients, 5% (7) of patients had inlay and hinge exchange due to hematoma or prolonged wound secretion, 2% (3) of patients had secondary patella revision and one patient each underwent revision for metallosis and periprosthetic fracture. All revisions were performed following revision TKA and none of the primary implants underwent revision surgery. There were no revision surgeries for loosening or instability.

### 3.2. Revision-Free Survival

**Figure 1:**
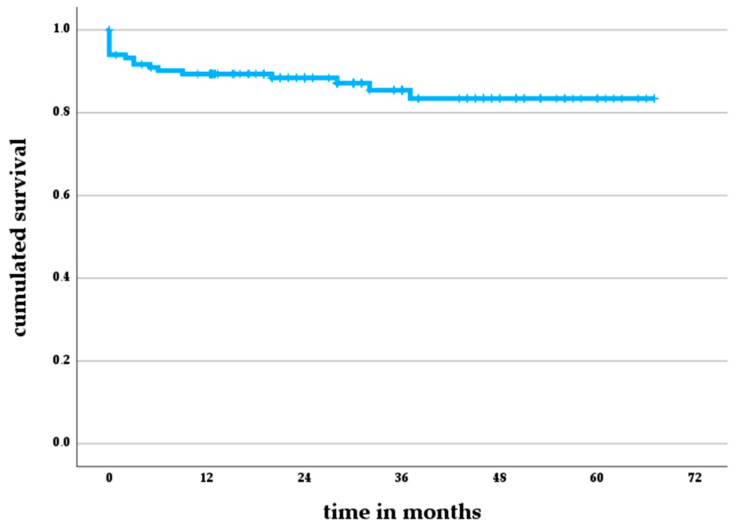
Revision-free implant survivorship according to the Kaplan–Meier method.

The revision-free implant survival amounted to 89% (95% CI 83–95%) after one year and to 83% (95% CI 75–91%) after five years (Figure 1).

### 3.3. Results after Two-Stage Exchange for PJI

In total, 8% (6/70) of patients underwent repeat implant removal surgery for chronic recurrent infection.

**Figure 2:**
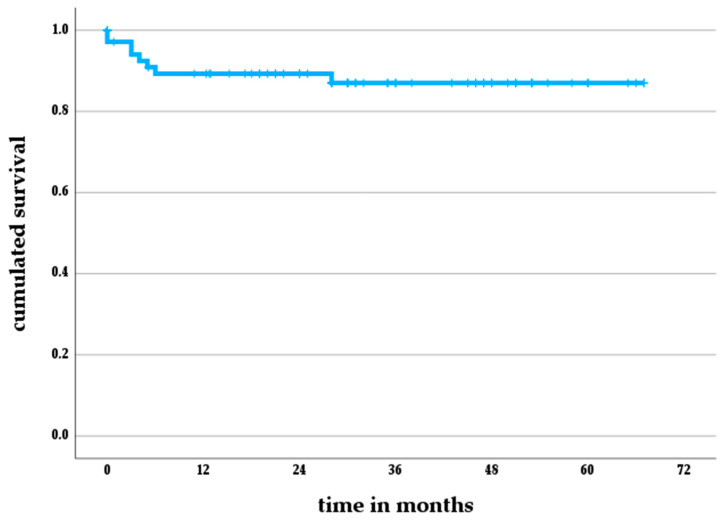
Re-infection-free implant survivorship according to the Kaplan–Meier method for patients who underwent two-stage revision for PJI and were reconstructed with the RHK TKA (70/133).

The survival free from revision for reinfection amounted to 89% (95% CI 82–96%) after one year and to 87% (95% CI 79–95%) after five years (Figure 2).

### 3.4. Risk Factors for Revision

We found that RHK implants used for primary TKA had better survivorship at five years compared to revision TKA (100% vs. 80% (95% CI 71–89%) (*p* = 0.05)). On the other hand, there was no difference in the five-year revision-free survivorship depending on the indication of revision surgery (PJI, loosening, instability, and fracture) (*p* = 0.69).

There was a higher number of previous surgeries in those patients who underwent re-revision surgery (2.5 (IQR 2–5) vs. 2 (IQR 1–3), *p* = 0.05). Although, there was no difference in five-year survival for patients with previous joint infection compared to those with aseptic previous surgery (78% (95% CI 64–91%) vs. 85% (95% CI 72–99%), *p* = 0.25).

With the numbers available, there was no difference in five-year revision-free survival and revision risk in general for various risk factors (Table 2 and Table 3).

### 3.5. Functional Outcome

The median Oxford Knee Score amounted to 33 (IQR 26–38) (available in 54% (72/133) patients), and the median range of motion amounted to 93° (IQR 90–110).

## 4. Discussion

Rotating-hinge implants have an important role in total knee arthroplasty as they provide the possibility to address instability, complex deformity or bone loss during complex primary or revision surgery [2,16,24,25,26,27]. Considering the expanding numbers of primary and particularly revision TKA in many healthcare systems, the need to utilize such implants is likely to grow [28,29]. However, as the reported results in terms of implant-associated complications are still higher compared to primary implants or implants with less constraint [6,10,30], there is an ongoing need for improvements in implant design in order to empower surgeons to react to individual anatomy or defects. This study investigates short-to-medium results of a novel RHK TKA system focusing on the likelihood of implant complications, the indications for potential revision surgery and functional outcome. Furthermore, as a majority of patients in the present study have undergone staged revision TKA for periprosthetic joint infection, reinfection-free survivorship was evaluated as well as potential risk factors for failure.

We found that while no patient with primary TKA using this novel implant RHK design underwent revision surgery, the overall revision rate was 13%, resulting in a revision-free survival probability of 17% after five years. The risk of reinfection was 13% after five years among the 70 patients included in the study who underwent a two-stage revision for chronic PJI. In this context, patients with a higher number of previous surgeries were more likely to undergo re-revision. In patients with compromised knee stability, bone loss or deformity, rotating-hinge implants are a useful and reasonable reconstructive approach, although even with improvements in implant design and novel implant features, the risk of complications and associated revision surgeries remains relatively high.

While, historically, fixed-hinge knee designs had a notoriously high rate of complications, there are several studies on the survival and outcome of modern RHK TKA designs. In a large study including around 400 implants of various RHK designs [9], Cottino et al. found the likelihood of revision surgery to be around 20% after five years which is similar to the results of the present study. While the reasons for revision were similar with periprosthetic infection being the leading cause, they found aseptic loosening to be the second most common reason for revision surgery which is contrary to this study’s observation. This is most likely due to the longer follow-up of their study as aseptic loosening might become more relevant with longer wear and repetitive strain on the implant. As the study by Cottino et al. has included a greater number of implants with a long-term follow up, the promising results of the present study with no revisions due to loosening of wear should be revisited with a longer follow up. Contrary to these findings, a study on 78 RHK TKA for primary and revision surgery by Kearns et al. [31] found that complications occurred in 38% of their patients leading to an implant survivorship of 70% after five years. While this rate is higher than in the present study, the authors noted a high percentage of concomitant extensor mechanism repairs were performed in their patients while the reasons for the use of a RHK design were otherwise similar. Although we did not include patients with dedicated extensor mechanism repair in the present study, it appears plausible that patients with compromised soft tissues are at increased risk for failure and revision surgery. Notably, Kearns et al. included eight patients who underwent muscle transfer and split skin coverage for soft tissue repair which has been known to be a high-risk procedure when performed for knee PJI [32].

The use of stems is a necessity in the presence of an RHK design in order to reduce the transmitted forces to the tibial plateau and, therefore, early loosening [1,2,33,34,35]. While this is generally accepted, optimal stem fixation is less clear with several studies investigating cemented versus uncemented stem designs [33]. The general approach in this study was to use hybrid fixation with an uncemented hydroxyl-apatite-coated stem and juxtaarticular cement fixation which, in the short term, led to no aseptic loosening. The use of an uncemented stem has the advantage that if implant removal is necessary during revision, the time-consuming and sometimes technically difficult task of cement removal can be avoided.

On the other hand, in cases of insufficient proximal force transmission, the end of stem pain may be more common with cortical hypertrophy as a radiological corresponding phenomenon. However, this issue has only been sporadically reported in the literature [36]. While one registry-based study found improved survival of hybrid fixation for revision TKA [37], other studies found no difference in survival between cemented and uncemented stems in revision TKA [38]. Nonetheless, surgeons must respect various factors when deciding which approach in terms of stem fixation is chosen such as deformity, incongruence between positioning of the plateau and diaphysial axis, previous fixation with repeat cementation leading to a higher rate of loosening, as well as patient factors [33].

The majority of reconstructions in this study have been performed following implant removal for periprosthetic joint infection. PJI is a leading cause of revision surgery in revision TKA and the optimal management of chronic infections is still debated [39,40,41]. RHK TKA implants are frequently employed as bone loss and instability are common following the necessary debridement as part of the revision surgery for PJI [24,39]. The risk of re-revision due to recurrent PJI as part of a two-stage revision approach for PJI using the single-design RHK implant in this study was 8% at mid-term follow-up. Farid et al., in a similar study using a different single-design RHK revision implant as part of a two-stage exchange for PJI in 60 cases, found a revision rate for recurrent infection of 18% which is comparable to the present study. However, one must acknowledge a longer follow-up time in their study; although, it has been suggested that a one-year follow-up after surgery for PJI suffices to declare infection eradication [42]. Furthermore, the slightly higher infection rate in their study might be due to the fact that they included distal femoral replacements with an RHK design that are known for their higher risk of reinfection when used as part of a two-stage exchange [41,43]. However, considering the small numbers of recurrent infections, even in large series, the underlying factors associated with re-revision remain widely unknown and future studies are needed [44,45].

While this is a single-center study that first investigates patients who underwent surgery with a novel, single-design RHK implant and managed to observe a reasonable number of patients, the present results must be interpreted acknowledging several limitations: Firstly, it is a retrospective study with the inherent drawbacks of such a design. Among these there are the reliance on follow-up data and completeness of electronic patient files. Therefore, as patients might have undergone surgery elsewhere, it is likely that the present results might be on the high end of survival estimates. Secondly, while the overall number of patients included allows for meaningful analysis, the number of patients who suffered a certain type of complications or presented with an individual potential risk factor is limited. Therefore, the analysis of complications is prone to sparse data bias and there is a need for larger, most likely multicenter studies to employ a multivariate approach to investigate potential risk factors for failure. Thirdly, while it is accepted that postoperative function with a RHK design TKA is inferior to that of well-functioning unconstrained TKA, the functional outcome is of paramount importance, particularly in patients undergoing primary TKA. Unfortunately, standardized functional outcome measurements using the Oxford knee score were only available in slightly more than half of the patients included at the latest follow-up. Considering that function might be impaired, particularly in complex revision cases, surgeons should discuss functional aspects and individual patient needs when planning the use of an RHK design TKA.

## 5. Conclusions

This novel single-design rotating-hinge knee system displayed satisfactory survivorship when used in revision TKA although recurrent infection after treatment for PJI remains an issue, particularly in patients with multiple previous surgeries. Contrary, patients with this implant used as a primary implant did not undergo revision surgery. Considering the expanding indications for TKA even in complex primary situations, RHK implants present a viable option. On the other hand, for multiply revised TKA, modern design RHK implants provide an option to restore a stable knee joint and reconstruct bone defects although the re-revision risk is higher.

## Figures and Tables

**Table 1:** Demographic factors.

Variable	Median (25–75% Interquartile Range)
age	72 (62–79)
BMI	30 (25–33)
CCI	4 (2–5)
ASA	2 (2–3)
previous revisions	2 (2–4)

BMI body mass index; CCI Charlson Comorbidity index; ASA American Society of Anesthesiologists.

**Table 2:** Risk factors for revision in survival analysis.

Variable	Five Year Survival Probability (95% CI)	*p*-Value (Log-Rank)
male vs. female	81% (95% CI 68–93%) vs. 86% (95% CI 78–96%)	0.09
smokers vs. non-smokers	95% (95% CI 86–100%) vs. 83% (95% CI 73–93%)	0.27
diabetics vs. non-diabetics	91% (95% CI 81–100%) vs. 83% (95% CI 70–94%)	0.53

**Table 3:** Risk factors for revision non-parametric comparative analysis.

Variable	Median (25–75 IQR)	*p*-Value
age	74 (IQR 67–78) vs. 72 (IQR 62–80)	0.63
BMI	31 (IQR 26–34) vs. 29 (IQR 25–33)	0.45
CCI	4 (IQR 3–5) vs. 4 (IQR 2–5)	0.69
ASA	2 (IQR 2–3) vs. 2 (IQR 2–3)	0.52

## Data Availability

Raw data are available upon reasonable request.
